# The relationship between the scorpion sting incidents that occurred in Bodrum, Turkey, and local climatic variables: A retrospective observational study

**DOI:** 10.1097/MD.0000000000048752

**Published:** 2026-05-15

**Authors:** Caner İşkorur, Halil Mutlu, Mustafa Korkut, Ali Avci, Ökkeş Zortuk, Nadir Şener

**Affiliations:** a Department of Emergency Medicine, Acibadem Bodrum Hospital, Muğla, Turkey; b Department of Emergency Medicine, Primary Health Care Corporation, Doha, Qatar; c Department of Emergency Medicine, Health Science University Antalya Training and Research Hospital, Antalya, Turkey; d Department of Emergency Medicine, Antalya City Hospital, Antalya, Turkey; e Department of Emergency Medicine, Bandirma Onyedi Eylül University, Balikesir, Turkey; f Department of Orthopedics and Traumatology, Acibadem Bodrum Hospital, Muğla, Turkey.

**Keywords:** climate change, climatic variables, scorpion stings, temperature

## Abstract

This study aimed to assess the relationship between scorpion sting incidents that resulted in emergency department (ED) visits in Bodrum, Muğla, located in the Aegean region, and local climatic variables, from 2015 to 2023. This retrospective, single-center observational study included scorpion sting incidents that resulted in ED visits between 2015 and 2023, along with meteorological data from the Bodrum district. Meteorological variables, including maximum and minimum air temperatures, relative humidity, and seawater temperature, from 2015 to 2023, were obtained from the Turkish State Meteorological Service. All temperature values were recorded in degrees Celsius (°C), relative humidity as percentage (%), and seawater temperature in degrees Celsius. The meteorological data were collected as annual average values calculated from daily measurements throughout each year. Years exceeding the regional mean temperature of 21.2°C were classified as high-temperature years; those below this threshold were classified as low-temperature years. The relationship between changes in meteorological parameters and scorpion sting incidents was demonstrated using Pearson correlation analysis. A total of 473 scorpion sting incidents were included in the analysis. In the comparison based on the average temperature value, the average prevalence rate in years with above-average temperatures was found to be 0.21% ± 0.052%, which was significantly higher than in years with below-average temperatures (0.14% ± 0.018%, *P* = .014). Both the minimum temperature in the Bodrum district (Pearson *r* = 0.7919, *P* = .011) and the average temperature (Pearson *r* = 0.878, *P* < .001) were positively correlated with scorpion sting incidence. A negative correlation was found between relative humidity and incidence (Pearson *r* = −0.695, *P* = .037). No significant correlation was observed between sea temperature and incidence. Hospitalization rates were positively correlated with both minimum and average temperatures. Rising local temperatures are associated with an increase in scorpion sting incidents and may serve as an exploratory indicator for monitoring trends in scorpion sting frequency. Additionally, when appropriate meteorological data are used, this can help determine the number of antivenom vials required in EDs. However, these findings are descriptive and exploratory and should be interpreted with caution. Larger datasets and more robust analyses are needed to confirm the observed associations.

## 1. Introduction

In Turkey, scorpion envenomation represents a significant public health concern, particularly in the Southeastern Anatolia region, where pediatric cases are frequently observed during the summer months.^[1]^ Scorpion envenomation represents a significant global health burden, with annual incidence approaching 1.2 million cases and mortality exceeding 3000 worldwide. Most scorpion envenomations result in mild local reactions similar to other arthropod stings and require only symptomatic treatment. However, around 30 species, predominantly from the *Buthidae* family, are recognized as medically important and capable of causing life-threatening envenomations in humans.^[2]^

In the Southeastern region of Turkey, *Androctonus crassicauda* is a predominant scorpion species causing envenomations in children, with clinical presentations ranging from mild local symptoms to severe systemic toxicity requiring intensive care.^[3]^ Young children are particularly prone to developing more severe envenomation syndromes due to the higher venom dose administered per unit of body weight.^[4]^ Although scorpions are found throughout Turkey, medical literature on scorpion envenomations remains limited. The most important health-threatening scorpion species in Turkey include *A. crassicauda*, *Leiurus quinquestriatus*, *Mesobuthus gibbosus*, and *Mesobuthus eupeus*, all belonging to the family *Buthidae*.^[5]^

As in other places, stings occur more frequently during the hot months, from May to September. Antivenom is commonly used in Turkey for severe scorpion envenomation. A monovalent antivenom is produced by administering *A crassicauda* venom to horses, followed by enzymatic digestion and purification of the serum. According to the national poisoning treatment protocol, hydrocortisone and antihistamines are commonly administered together. The inappropriate use of antivenom and antihistamines in the management of scorpion stings has been documented, highlighting the need for adherence to evidence-based treatment protocols and updated clinical guidelines.^[6]^

Scorpion stings are more frequently reported during the summer months and predominantly occur in rural areas, where access to healthcare facilities may be limited.^[1,3]^ The human impact on the climate system is clear, and recently, human-caused greenhouse gas emissions are at their highest levels in history. Recent climate changes have had widespread effects on both humans and natural systems. Global temperatures have shown a consistent warming trend over recent decades. Climate variables, including temperature and precipitation patterns, have been identified as predictive indices for the frequency of scorpion stings in endemic regions.^[7]^ Climate change has been responsible for a significant and increasing disease burden, with vulnerable populations, including children, experiencing disproportionate health impacts from rising temperatures and extreme weather events.^[8]^

A literature review revealed the scarcity of research on the relationship between temperature trends and scorpion sting incidence in Turkey. Therefore, this study examined the relationship between local temperature trends and emergency department (ED) visits for scorpion stings in Bodrum, Muğla, from 2015 to 2023.

## 2. Materials and methods

This study was approved by the Acibadem Mehmet Ali Aydinlar University Medical Research Ethics Committee (approval number: 2024-17/651, date: October 31, 2024). Due to the retrospective nature of the study using anonymized patient data, informed consent was waived by the ethics committee.

This retrospective single-center observational study included all pediatric and adult patients with scorpion stings who presented to the secondary-level ED in Bodrum, Muğla, Turkey, from 2015 to 2023. Data were extracted from the hospital electronic medical record system by investigators. Cases were identified using International Classification of Diseases codes X22 (contact with scorpions) and T63.2 (toxic effect of scorpion venom), and each case was subsequently verified through individual medical record review. A total of 484 cases were initially identified during the study period. Eleven patients were excluded: 9 due to incomplete or missing medical records and 2 due to misidentified arthropod exposures (1 centipede bite and 1 scolopendra sting). The remaining 473 verified scorpion envenomation cases were included in the final analysis.

### 2.1. Clinical classification and management

All cases were retrospectively reviewed through medical record examination, and clinical severity grading was assigned based on the documented findings. Scorpion sting cases were classified according to severity grading as follows:

Grade 1: mild pain at the sting site with no systemic manifestations.
Grade 2: severe pain extending beyond the affected extremity, with severe pain on palpation (positive tapping/percussion test).
Grade 3: somatic neuromuscular manifestations, including extremity jerks, hypertension, sweating, involuntary tremors, cranial nerve involvement (blurred vision, impaired eye movements, salivation, fasciculation, dysphagia).
Grade 4: somatic and neuromuscular manifestations along with serious complications such as myocardial infarction, pulmonary edema, convulsions, and shock.
Tapping/percussion test: increased pain on light tapping over the sting site.

All treatments administered were standardized according to this clinical staging system. Antivenom (antiserum) administration was performed according to the severity of envenomation (grades 2–4). No supply issues were encountered regarding the availability of antivenom in the hospital throughout the study period.

Hospitalization criteria included grade 2 or higher envenomation, pediatric patients with or without systemic symptoms, and patients requiring antivenom administration.

Daily data on maximum and minimum air temperatures, relative humidity, and seawater temperature for Bodrum, Muğla, from 2015 to 2023 were sourced from the Turkish State Meteorological Service database. All temperature values were recorded in degrees Celsius (°C), relative humidity as a percentage (%), and seawater temperature in degrees Celsius. The meteorological data were collected as annual average values calculated from daily measurements throughout each year. The threshold of 21.2°C represents the overall mean annual temperature across the 9-year study period. This value was used to classify study years – not individual days or months – into high-temperature (≥21.2°C) and low-temperature (<21.2°C) categories.

Incidence and prevalence measures were calculated to quantify the burden of scorpion stings in our ED. The annual incidence rate was expressed as the number of scorpion sting cases per 100,000 ED visits, calculated by dividing the yearly scorpion sting cases by the total number of ED visits in that year and multiplying by 100,000. This ED visit-based incidence rate provides a standardized measure that accounts for variations in healthcare utilization over time. Cumulative incidence over the 9-year study period (2015–2023) was calculated as the total number of scorpion sting cases (n = 473) divided by the total ED visits during the entire study period (n = 294,893) and expressed per 100,000 visits. Period prevalence was calculated as the proportion of scorpion sting-related ED visits among all ED admissions during the study period, expressed as a percentage.

### 2.2. Statistical analysis

The combined meteorological and case dataset was analyzed with Statistical Package for the Social Sciences 27 (International Business Machines Corporation), and graphs were generated using GraphPad Prism 9. After data classification, categorical data were presented as frequencies and percentages, and distribution analysis was conducted for numerical data. Normally distributed data were expressed as mean ± standard deviation (SD), whereas non-normally distributed data were reported as median and interquartile range. Parametric tests were applied to normally distributed numerical variables, whereas non-normally distributed variables were compared using the Mann–Whitney *U* test. Pearson correlation was used to evaluate the association between meteorological variables and case numbers. Values with a *P*-value below .05 were considered statistically significant.

## 3. Results

A total of 473 scorpion sting cases were analyzed; the mean patient age was 42.52 ± 20.16 years (mean ± SD, n = 473). Of the total study population, 244 patients (48.7%) were male. During the study period, scorpion sting presentations averaged 52.55 ± 15.62 per year in the ED. Over the study period, Bodrum district recorded a mean ± SD minimum temperature of 16.68 ± 0.75°C, a mean ± SD maximum of 25.72 ± 0.22°C, and an overall mean of 21.20 ± 0.41°C.

Table 1 shows the annual number of cases and temperature changes.

**Table 1:** Annual distribution of scorpion sting cases, emergency department visits, incidence rates, prevalence, meteorological parameters, severity grades, and antiserum administration in Bodrum district (2015–2023).

Years	Temperature			Total ED visits (n)	Scorpion sting cases (n)	Incidence rate*	Grade ^†^				Hospitalization	Antiserum	Prevalence %
	Maximum	Minimum	Mean				1	2	3	4			
2015	25.57	15.68	20.63	27,758	35	126.1	31	4	0	0	2	0	0.126
2016	25.92	15.98	20.95	32,123	41	127.6	39	2	0	0	1	0	0.128
2017	25.85	16.04	20.95	34,212	41	119.8	39	2	0	0	0	0	0.120
2018	25.63	16.82	21.22	40,450	51	126.1	51	1	0	0	0	0	0.126
2019	25.43	16.60	21.01	37,667	45	119.5	43	2	0	0	1	0	0.119
2020	25.98	17.09	21.53	27,455	60	218.5	57	1	1	1	3	2	0.219
2021	25.98	17.22	21.60	31,346	81	258.4	75	4	2	0	4	2	0.258
2022	25.43	16.63	21.03	33,104	46	138.9	45	0	1	0	2	2	0.139
2023	25.76	18.14	21.95	30,778	73	237.2	43	22	8	0	13	35	0.237

Each grade represents different severity levels of envenomation as defined in the materials and methods section.

TAP = tapping/percussion.

* Incidence rate: per 100,000 emergency department visits.

† Grade classification: Grade 1: mild pain at the sting site with no systemic manifestations. Grade 2: severe pain extending beyond the affected extremity, with severe pain on palpation (positive TAP test). Grade 3: somatic neuromuscular manifestations, including extremity jerking, involuntary tremors, cranial nerve involvement (blurred vision, impaired eye movements, salivation, fasciculation, dysphagia). *In the scorpion species endemic to our region, manifestations such as hypertension, sweating, tremors, and chest pain are more prominent. Grade 4: somatic and neuromuscular manifestations along with serious complications such as myocardial infarction, pulmonary edema, convulsions, and shock.

The overall cumulative incidence of scorpion stings was 160.4 per 100,000 ED visits over the 9-year study period (Table 1). Annual incidence rates showed considerable variation, ranging from 119.5 per 100,000 ED visits in 2019 to 258.4 per 100,000 ED visits in 2021. A fluctuating pattern with intermittently elevated rates was observed from 2020 onwards, with incidence rates of 218.5, 258.4, 138.9, and 237.2 per 100,000 ED visits in 2020, 2021, 2022, and 2023, respectively. The overall period prevalence of scorpion stings among all ED admissions was 0.160% (473 out of 294,893 total ED visits during the study period).

**Figure 1.**
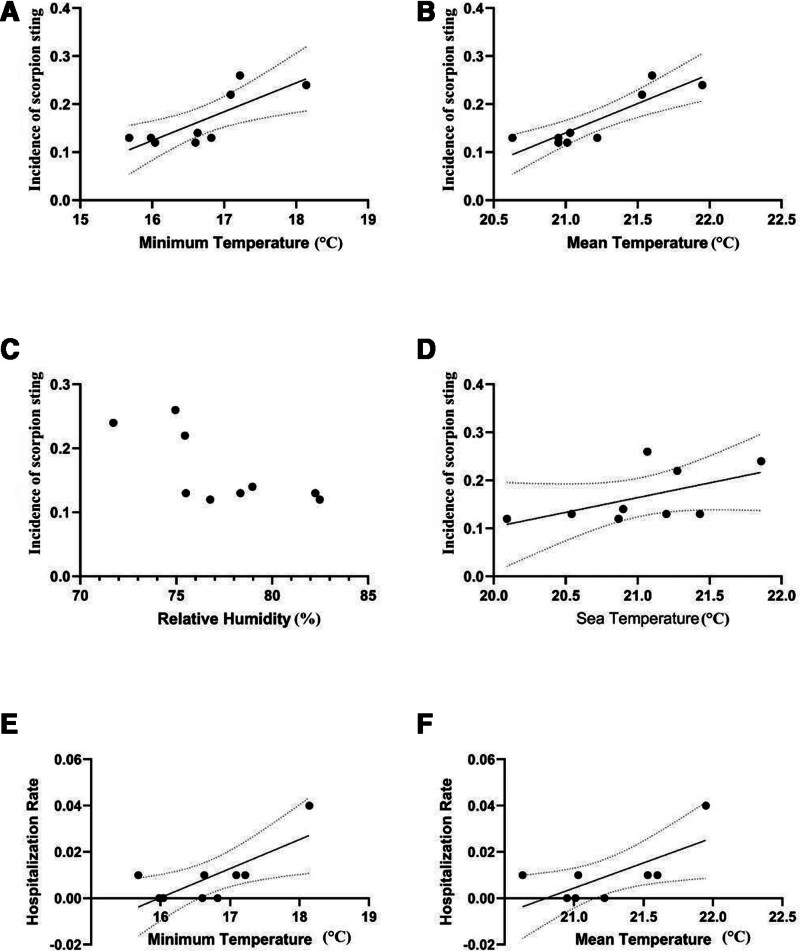
Correlation between meteorological parameters and scorpion sting incidence and hospitalization rates in Bodrum district (2015–2023). (A) Correlation between minimum temperature and incidence, (B) correlation between mean temperature and incidence, (C) correlation between relative humidity and incidence, (D) correlation between seawater temperature and incidence, (E) correlation between minimum temperature and hospitalization rate, and (F) correlation between mean temperature and hospitalization rate.

Based on the overall study-period mean temperature of 21.2°C, years above this threshold were classified as high-temperature years and those below as low-temperature years. Years with mean temperatures above 21.2°C showed a prevalence of 0.21% ± 0.05%, which was significantly greater than in years below this threshold (*P* = .014). In Table 2, prevalence, hospitalization, and case numbers are compared according to mean temperature value. Minimum temperature in the Bodrum district was positively correlated with scorpion sting incidence (Pearson *r* = 0.79, *P* = .011; Fig. 1A), as was average temperature (Pearson *r* = 0.88, *P* < .001; Fig. 1B). Relative humidity was significantly negatively correlated with scorpion sting incidence (Fig. 1C), whereas sea surface temperature showed no significant correlation with sting incidence (Fig. 1D). A positive correlation was identified between hospitalization rate and minimum temperature (Fig. 1E) as well as average temperature (Fig. 1F). Table 3 shows the correlation between scorpion sting incidence and hospitalization rates with meteorological values.

**Table 2:** Comparison of scorpion sting prevalence, hospitalization rates, and case number stratified by average annual temperature categories (low: <21.2°C vs high: ≥21.2°C) in Bodrum district during 2015 to 2023. Values are presented as mean ± standard deviation. *P*-values were calculated using the Mann–Whitney U test.

	Low (n = 4)*	High (n = 5)*	*P*-value
Prevalence	0.12 ± 0.007	0.21 ± 0.052	.014
Hospitalization rate	0.0031 (0.01)	0.0118 (0.03)	.190
Case number	41.60 ± 4.33	66.25 ± 13.35	.006

Statistical comparison demonstrates significantly higher prevalence in high-temperature years.

* Temperature category: low (<21.2°C average annual temperature) versus high (≥21.2°C average annual temperature).

**Table 3:** Pearson correlation coefficients (*r*) and statistical significance (*P*-values) between meteorological parameters and both scorpion sting incidence and hospitalization rates in Bodrum district over the study period (2015–2023).

Incidence, hospitalization rate	Meteorological parameters	*r*	95% CI	*R* ^2^	*P*-value
Incidence	Minimum temperature (°C)	0.7919	0.2696–0.9542	0.6271	.011
	Maximum temperature (°C)	0.8784	0.5144–0.9742	0.7717	<.001
	Relative humidity (%)	−0.7121	−0.9344 to −0.09107	0.5071	.015
	Sea temperature (°C)	0.5514	−0.1778 to 0.8897	0.3041	.061
Hospitalization rate	Minimum temperature (°C)	0.7428	0.1553–0.9422	0.5518	.011
	Mean temperature (°C)	0.7029	0.07269–0.9320	0.4940	.017

Positive correlations indicate parameters that increase together, while negative correlations indicate inverse relationships.

CI = confidence interval.

## 4. Discussion

The study demonstrated that scorpion sting incidents in Bodrum, Muğla, Turkey, showed a significant positive correlation with rising temperatures between 2015 and 2023. Bodrum is located in the Aegean region of Turkey. The average prevalence rate in years with above-average temperatures (0.21% ± 0.052%) was significantly higher than in years with below-average temperatures (0.14% ± 0.018%, *P* = .014). With 473 cases documented over 9 years (cumulative incidence: 0.160%), this study represents one of the largest datasets on scorpion envenomation in the Aegean region. Additionally, a negative correlation was found between relative humidity and scorpion sting incidence, while hospitalization rates also increased with rising temperatures. These findings suggest that climate change may be associated with increased scorpion activity and subsequent human encounters.

In recent years, increasing temperatures have been associated with higher incidence of scorpion stings in endemic regions, with climatic variables serving as predictive indicators for envenomation risk.^[9,10]^ Environmental factors, particularly ambient temperature, have been shown to influence human-wildlife encounters and increase the risk of envenomation from various species, including snakes and venomous arthropods.^[11]^

In Duarte et al’s study, no significant correlation was found between scorpion incidence changes and the percentage of urban areas. Due to climatic conditions, scorpion incidents exhibited a wide distribution, particularly in tropical and subtropical regions; therefore, urbanization was not the main factor contributing to the increase in scorpion incidents.^[12]^ It is stated that climatic conditions may have a greater impact compared with urban growth. However, it is important to note that urbanization and climate change may have interconnected effects. Urban heat island effects can exacerbate temperature increases in already warming regions, potentially creating more favorable conditions for scorpion activity. Future studies should consider the combined influence of these factors.

In 2005, Chowell et al found that scorpion sting frequency in Colima, Mexico, was significantly correlated with temperature and rainfall, reflecting the region’s pronounced seasonal pattern.^[13]^ The geographic distribution of venomous species is changing as a result of ongoing climatic shifts, with climate warming facilitating range expansions in many arthropod species through evolutionary adaptations at range margins that potentially accelerate distributional shifts via positive feedback mechanisms.^[14–16]^ Rising temperatures have led to marked shifts in species’ distributions, activity periods, and rates of human contact. Our study found a positive correlation between scorpion sting incidence and rising average temperature, a key indicator of climate change. These exploratory findings may inform future efforts to monitor seasonal scorpion sting patterns and develop preventive strategies. Consistent with our findings, multiple studies from different geographic regions have demonstrated significant positive correlations between ambient temperature and scorpion sting incidence. In southern Iran, Ebrahimi et al reported a positive correlation with temperature (*P* < .001) and a negative correlation with relative humidity (*P* = .002).^[17]^ Similarly, Hurtado-Díaz et al found a 9.8% increase in scorpion sting cases per 1°C temperature increase in the hottest regions of Mexico, with minimum temperatures exerting a delayed effect that varied by climatic region.^[10]^ Comparable associations were also reported in Algeria and Iran, where temperature, evaporation, and sunshine hours were positively correlated with scorpion sting cases, while relative humidity and rainfall showed inverse relationships.^[7,18]^ These findings collectively support our observation that scorpion sting incidents have a negative correlation with relative humidity. In our study, no correlation was found between seawater temperature and scorpion sting incidents. Predictive models by Selmane et al suggested that a 1-degree increase in temperature would result in an increase of 0.18 scorpion stings per 100,000 people per month.^[18]^ Increasing temperatures and drought conditions associated with climate change in the Mediterranean basin are contributing to enhanced scorpion activity. In Turkey, this is particularly evident in regions such as Southeastern Anatolia (30.4%), the Aegean (23.5%), and the Mediterranean (24.9%), where scorpion sting incidence peaks during summer and spring months.^[16,19]^ In Bodrum, Aegean region, higher summer temperatures and increased outdoor activities boost scorpion activity and elevate sting risk. Climate change is expected to shift the geographical ranges of scorpions and other venomous species. For example, the correlation between snakebite incidents and temperature increase indicates that the biological activity of snakes and other venomous animals also increases in hot climates. A study conducted in Georgia, United States, noted that each 1°C increase in maximum temperature was associated with a 5.6% increase in venomous snakebite ED visits. This relationship may be attributed to both the increased activity of ectothermic snakes in warmer temperatures and the rise in outdoor activities among people.^[11]^ Species distribution models under climate change scenarios predict northward range shifts and expansion into new regions for medically important scorpion species. When evaluated for Turkey, similar distributional changes and increased scorpion activity in currently endemic regions can be anticipated due to the effects of climate change.^[20]^

Rising temperatures and changing climatic conditions are associated with increased contact between people and venomous creatures. Cases of venomous animal exposure represent a substantial public health risk. Changes in the geographic distribution of these animals due to climate change may present greater challenges for public health systems in the future.

### 4.1. Limitations

Our study has some limitations. Bodrum’s population fluctuates dramatically between winter and summer due to seasonal tourism, which may independently increase ED presentations regardless of meteorological conditions. The coronavirus disease 2019 outbreaks of 2020 and 2021 triggered a significant relocation of urban dwellers to Bodrum. Additionally, urbanization patterns, environmental changes, and healthcare-seeking behavior may have influenced the number of ED visits independently of climatic factors. These potential confounding variables could not be adjusted for in the current analysis and should be considered when interpreting the results. Furthermore, not all individuals stung by scorpions may seek medical care at EDs, particularly those experiencing only mild local symptoms, which may lead to an underestimation of the true incidence of scorpion stings in the region. Additionally, this study represents one of the largest datasets collected over 9 years from the Bodrum district in the Aegean region. The correlation analyses were based on annually aggregated data, resulting in only 9 data points per variable, which limits statistical power. More granular temporal analyses using monthly or seasonal data with count-based regression models (e.g., Poisson or negative binomial regression) could provide a more robust analytical framework and should be considered in future studies. The findings should therefore be interpreted as associational and exploratory rather than predictive or causal.

## 5. Conclusion

In conclusion, rising local temperatures were associated with more frequent scorpion stings in Bodrum, Muğla, suggesting that climatic variables may play a role in scorpion sting incidence. Additionally, scorpion sting intensity can be anticipated using meteorological indicators. In light of this, health authorities should implement tailored summer prevention plans in high-risk areas to lower scorpion sting incidents. In hot regions most affected by climate change, emergency health services must prepare for scorpion sting incidents by ensuring an adequate antivenom supply, as this is of critical importance for public health. However, it is important to acknowledge that these findings are descriptive and exploratory in nature and should be interpreted with caution. Larger datasets spanning longer time periods, combined with more robust analytical approaches, including multivariate analyses and time-series methods, are needed to confirm the observed associations and better understand the complex relationship between climate change and scorpion sting incidents. Further research involving more comprehensive studies in different geographical regions would be beneficial.

## Acknowledgments

We greatly appreciate all of the study participants.

## Author contributions

**Conceptualization:** Caner İşkorur, Halil Mutlu, Mustafa Korkut, Ali Avci, Nadir Şener.

**Data curation:** Caner İşkorur, Halil Mutlu, Mustafa Korkut, Ali Avci, Ökkeş Zortuk, Nadir Şener.

**Formal analysis:** Caner İşkorur, Halil Mutlu, Mustafa Korkut, Ali Avci, Ökkeş Zortuk.

**Funding acquisition:** Caner İşkorur.

**Investigation:** Caner İşkorur, Halil Mutlu, Mustafa Korkut, Ali Avci, Ökkeş Zortuk, Nadir Şener.

**Methodology:** Caner İşkorur, Halil Mutlu, Mustafa Korkut, Ali Avci, Nadir Şener.

**Supervision:** Caner İşkorur, Mustafa Korkut, Nadir Şener.

**Validation:** Mustafa Korkut, Ökkeş Zortuk, Nadir Şener.

**Writing – original draft:** Caner İşkorur, Halil Mutlu, Mustafa Korkut, Ali Avci, Ökkeş Zortuk, Nadir Şener.

**Writing – review & editing:** Caner İşkorur, Halil Mutlu, Mustafa Korkut, Ali Avci, Ökkeş Zortuk, Nadir Şener.
